# Association between fasting insulin and C-reactive protein among adults without diabetes using a two-part model: NHANES 2005–2010

**DOI:** 10.1186/s13098-021-00645-4

**Published:** 2021-03-10

**Authors:** Amanda L. Missel, Laura R. Saslow, Dina H. Griauzde, Donna Marvicsin, Ananda Sen, Caroline R. Richardson, Xuefeng Liu

**Affiliations:** 1 Department of Learning Health Sciences, Medical School, University of Michigan, 1160 NIB 300, Ann Arbor, MI 48104 USA; 2 Department of Health Behavior and Biological Sciences, School of Nursing, University of Michigan, Ann Arbor, MI USA; 3 Internal Medicine, Medical School, University of Michigan, Ann Arbor, MI USA; 4 VA Ann Arbor Healthcare System, Ann Arbor, MI USA; 5 Family Medicine, Medical School, University of Michigan, Ann Arbor, MI USA; 6 Department of Biostatistics, School of Public Health, University of Michigan, Ann Arbor, MI USA; 7 Systems, Populations and Leadership, University of Michigan, Ann Arbor, MI USA

**Keywords:** Inflammation, Diabetes, Insulin, CRP

## Abstract

**Introduction:**

Chronic inflammation is associated with the development, progression and long-term complications of type 2 diabetes. Hyperglycemia is associated with chronic low-grade inflammation, and thus has become the focus of many screening and treatment recommendations. We hypothesize that insulin may also be associated with inflammation and may be an additional factor to consider in screening and treatment.

**Methods:**

This study used National Health and Nutrition Examination Survey data from 2005 to 2010 to analyze the association between fasting insulin and C-reactive protein (CRP). A two-part model was used due to the high number of values reported as 0.1 mg/L. Two models were analyzed, both with and without the addition of waist circumference to other covariates in the model.

**Results:**

The final sample included 4527 adults with a mean age of 43.31 years. In the first model, higher fasting insulin was associated with increased odds of CRP > 0.1 mg/L (OR = 1.02, p < .001) and with higher CRP (β = 0.03, p < .001). In the adjusted model, including waist circumference as a covariate, higher fasting insulin was not associated with CRP > 0.1 mg/L (OR = 1.00, p = .307) but the association between higher fasting insulin and higher continuous CRP remained significant (β = 0.01, p = .012).

**Conclusion:**

This study found that higher fasting insulin is associated with higher CRP. These results suggest that treatment approaches that simultaneously decrease insulin levels as well as glucose levels may provide additive anti-inflammatory effects, and therefore may improve long-term outcomes for adults with type 2 diabetes.

Type 2 diabetes (T2DM) affects over 30 million U.S. adults and an additional 88 million U.S. adults are estimated to have prediabetes [1]. A growing body of literature demonstrates that inflammation is involved in the development, progression and long-term complications of type 2 diabetes (T2DM), and visceral adipose tissue and proinflammatory cytokines play a key role [2–4]. Visceral adipose tissue (i.e., fat stored in the abdominal cavity) promotes more inflammation than fat in other locations (e.g., subcutaneous tissue) [3, 4]. It is metabolically active and attracts macrophages, which then secrete proinflammatory cytokines [3, 4].

Hyperinsulinemia [5–7], defined by insulin secretion higher than normal relative to blood glucose levels [8], may be one proinflammatory factor associated with numerous ill health effects including heart disease [9–11] and stroke [12]. CRP (using a log transformation to approximate a normal distribution) was associated with the markers of insulin resistance and fasting insulin among a nondiabetic population, and the relationship was attenuated after controlling for waist circumference [13]. A positive association between fasting insulin and hs-CRP was also reported in a population not taking glucose-lowering medications [7] and between fasting insulin and CRP among nondiabetic females [6]. Additionally, an association between insulin and inflammation was observed in populations with T2DM and hyperglycemia [14].The mechanism by which hyperinsulinemia promotes inflammation is not clearly defined but may be related to its positive effect on body weight and visceral adiposity [15–17]. However, strategies to support a relative reduction in serum insulin levels may reduce inflammation and its health consequences, including prediabetes and T2DM.

Strategies to reduce serum insulin levels may include weight loss, dietary modifications (e.g., carbohydrate restriction, intermittent fasting) or use of certain anti-hyperglycemic agents among patients with T2DM. Such strategies may align with the American Diabetes Association’s latest clinical practice guidelines, which support a paradigm shift in T2DM management away from a “one-size-fits all” glucocentric treatment towards a more nuanced, patient-centered approach, which aims to mitigate adverse cardiovascular events. Glycemic control is more effective at preventing microvascular complications such as retinopathy, nephropathy, and neuropathy than macrovascular outcomes such as cardiovascular disease [18]. Understanding the relationship between insulin and inflammation may have important implications for the treatment and prevention of T2DM, especially because many of the pharmaceutical treatments for T2DM increase insulin levels through endogenous or exogenous means [19]. This is addressed in the new guidelines, which favor newer medications shown to reduce vascular complications over older agents [20].

The proinflammatory features of hyperglycemia make it difficult to separate the effects of increasing insulin from its glucose-lowering effects in participants with T2DM. Additionally, insulin levels vary with T2DM duration and treatment, partly due to hyperglycemia, beta cell failure, and glucose-lowering medications. For these reasons it is difficult to discern the relationship between insulin and the inflammatory CRP marker in a population with T2DM. The relationship between insulin and inflammation independent of visceral adipose tissue remains unclear. The present study will extend previous findings by utilizing differing methodology to account for the large number of near zero values, a larger sample, and more recent cycles of NHANES data. Finally, we will extend previous research by using a two-part model that accounts for the semi-continuous data to provide a post-estimation prediction of the actual difference in CRP based on tertiles of fasting insulin to estimate the clinical significance.

## Objective

The objective of the present study was to examine the independent association between fasting insulin and the inflammatory marker CRP in individuals without diabetes. In addition, we examined the role of visceral adiposity in this relationship.

## Methods

Data on CRP, fasting insulin, obesity-related measurements and other characteristics were collected from the National Health and Nutrition Examination Survey (NHANES) 2005–2010, conducted by the National Center for Health Statistics (NCHS) in the Centers for Disease Control and Prevention. All the participants signed written consent, and the surveys were approved by the NCHS Institutional Review Board. Interviews and physical examinations were completed on a nationally representative sample of non-institutionalized individuals in the United States. A multi-stage sampling design with weighted domains was used to provide national estimates. Details of the sampling design and guidelines for the NHANES study have been described elsewhere [21].

### Participants

To address the issues related to insulin and glucose levels in participants with T2DM noted above, our sample included adults aged 20 years and older without a diagnosis of diabetes. While we did not exclude anyone based on an upper age limit, NHANES reported the age of all individuals aged 85 years and older as age 85 for the 2005–2006 cycle. This was decreased to all individuals aged 80 and over reported as age 80 for 2007–2010 cycles. This allows them to ensure anonymity of participants due to fewer individuals in higher age groups. To reduce the bias in examining the independent associations between fasting insulin and CRP, and make our analysis consistent with previous NHANES surveys, we excluded individuals younger than 20 years of age, those who have a self-reported diagnosis of diabetes, use glucose-lowering medications, use cholesterol-lowering medications (having an anti-inflammatory effect) [22], or who were pregnant (due to changing physiology and waist circumference in pregnancy). The diabetes questionnaire provided information on the use of glucose-lowering medications or insulin injections.

### Measures

#### CRP

High-sensitivity C-reactive protein (hs-CRP) is an acute-phase reactant produced in the liver and a sensitive marker of low-grade inflammation [23]. Detection of CRP was done by latex-enhanced nephelometry using a Siemens/Behring Nephelometer, which allowed a conversion of the intensity of reflected light to milligrams of CRP per deciliter (mg/dL). The lowest reportable value was 0.02 mg/dL, everyone at or below the detection limit had a value reported as 0.01 mg/dL. Results were converted to mg/L to provide consistency with AHA risk categories. Equipment or reporting changes were not made during the three cycles of data used. Chronic conditions such as arthritis and smoking may raise the levels of CRP [24].

#### Fasting insulin

Only subjects who fasted overnight and were tested in the morning session were included in the study. Insulin was detected in picomoles per liter (pmol/L) with the Merocodia Insulin ELISA, in late 2009 it was changed to the Roche chemiluminescent immonoassay using the Elecsys 2010 analyzer [25].

#### Fasting glucose

This test was completed on refrigerated serum. During the 2007–2010 cycles, plasma was used on the Hexokinase-mediated reaction Roche/Hitachi Modular P Chemistry Analyzer. The 2005–2006 results were completed on the Roche/Hitachi 911 Analyzer. Results were reported in millimoles per liter (mmol/L) [26–28].

#### Hemoglobin A1c (HbA1c)

This test was completed on whole blood samples collected in an EDTA tube. During 2005–2006, the Toshoh A1c 2.2 Plus Glycohemoglobin Analyzer was used. A change was made 6 months into the 2007–2008 cycle to the G7 HPLC Glycohemoglobin Analyzer, which was used through 2010 [29–32].

#### Waist circumference

Waist circumference was measured with a flexible measurement tape in centimeters by trained health technicians. The anatomical landmark was the lateral border of the right ilium. A cosmetic pencil was used to note this landmark just above its uppermost lateral border.

#### Physical activity status

Physical activity status was established using the Global Physical Activity Questionnaire (GPAQ) [33]. This scale has been validated against accelerometer measurements. There was a moderate correlation between the self-reported questionnaires and accelerometer data with a wide interquartile range [34]. To reduce recurring small changes in surveys over the years, select survey questions/variables were dichotomized into subjects who participated in moderate or vigorous physical activity, or reporting no moderate or vigorous physical activity. These variables were then merged to create one dichotomous variable indicating whether the participant self-reported moderate or vigorous physical activity.

#### Smoking status

Smoking status was dichotomized into current smoker or non-smoker. Former smokers were categorized as non-smokers.

#### Demographic and socioeconomic factors

Age in years (analyzed as a continuous variable), race and ethnicity (non-Hispanic White/non-Hispanic Black/Other/Mexican or Hispanic), gender, and poverty-income ratio were obtained for each participant. Poverty-income ratio is a measure used by NHANES to provide an index of the ratio of family income to poverty. Family household income is used to calculate this measure. Families are defined as those residing together and related by birth, marriage, or adoption. Their total income from all sources, including wages, retirement income, disability payments, interest income, and assistance programs is used. Poverty level, set by the Department of Health and Human Services (HHS) poverty, are guidelines issued each year to qualify for many assistance programs. The ratio was then calculated by the ratio of total family income to poverty level. NHANES reports any value greater than 5 as 5, and a higher index indicated higher income. The value is reported as missing if family income is not reported [35].

### Statistical analyses

Summary characteristics provide nationally representative estimates and are adjusted for the complex sampling design. Continuous variables that have highly skewed distributions are described using the median and interquartile range. Unadjusted estimates were analyzed using a generalized linear model (GLM) with a gamma distribution and identity link function and with the appropriate fasting sample weights.

The main objective of the study is to model the association between fasting insulin and C-Reactive Protein (CRP). The CRP distribution was characterized by a significant spike at 0.1 mg/L. The values above 0.1 mg/L had a highly right skewed distribution. Values reported as 0.1 mg/L were recoded as zero for regression model purposes. A two-part model was employed to analyze the zero-inflated semi-continuous data. The two-part model is a mixture of two components; one that models the likelihood of zeros (typically a logistic regression model) and the other models the distribution of the non-zero part of the data. In our case, the non-zero part is modeled as a gamma distribution and identity link function to conform to the heavy skewness.

Two adjusted models were developed. The first model controlled for age, race, gender, smoking status, physical activity, and poverty-income ratio, but did not control for waist circumference. The second model controlled for waist circumference and the covariates of the first model. These models tested the hypothesis that there was an independent association between fasting insulin and CRP, after controlling for visceral adipose tissue (as reflected in waist circumference). The model also provides the predicted CRP for each level of all categorical variables to aid in quantifying the clinical relevance. Adjusted two-part models use males, non-Hispanic White race, non or former smokers, and no physical activity as the reference groups. All analyses were performed using Stata version 16, College Station, TX.

## Results

The final sample included 4527 adults with a mean age of 43.31 years, 49.03% of them male, 12.53% of Mexican or other Hispanic ethnicity, 70.52% of non-Hispanic White, 10.73% of non-Hispanic Black, and 6.22% who identified as another race or multi-racial. Their mean BMI characterized them as overweight, and their fasting insulin was at the high end of normal relative to their normal blood glucose. The summary characteristics are presented in Table 1.

**Table 1:** Summary characteristics among adults without diabetes

Variable	N = 4527
Categorical, N in millions (%)	
Sex	
Male	75.43 (49.03)
Female	78.39 (50.97)
Race	
Non-Hispanic White	108.48 (70.52)
Mexican/other Hispanic	19.27 (12.53)
Non-Hispanic Black	16.50 (10.73)
Other race/multi	9.57 (6.22)
Smoking	
Non/former smoker	117.81 (76.59)
Current smoker	36.01 (23.41)
Physical activity	
No	37.59 (24.44)
Yes	116.23 (75.56)
Continuous, mean (SE) or median (IQR) based on distribution	
Age years, mean (SE)	43.31 (0.40)
Poverty-income ratio, Mean (SE)	3.06 (0.04)
Waist circumference cm, mean (SE)	96.08 (0.38)
Weight kg, mean (SE)	80.88 (0.38)
Body mass index kg/m^2^, mean (SE)	28.01 (0.13)
Fasting glucose mmol/L, mean (SE)	5.48 (0.02)
Hemoglobin A1c %, mean (SE)	5.35 (0.01)
Fasting insulin pmol/L, median (IQR)	56.04 (36.00, 90.84)
CRP mg/L, median (IQR)	1.5 (0.60, 4.00)

Mexican/other Hispanic: Mexican or other Hispanic ethnicity; other race/multi: other race or Multiracial; SE: standard error

Median and interquartile range (IQR) used for highly right skewed distributions

National estimates based on complex survey design

There was a significant unadjusted association between fasting insulin and CRP (β = 0.03, CI 0.02, 0.03, p < .001) which remained after adjusting for all covariates except waist circumference in the first two-part model. Unadjusted estimates are presented in Table 2.

**Table 2:** Unadjusted associations between covariates and CRP

Covariate	β (p-value)
Age, years	0.06 (< .001)*
Sex	
Male	0.0 (Reference)
Female	1.07 (< .001)*
Race	
White	0.0 (Reference)
Mexican/Hispanic	− 0.01 (.959)
Black	0.57 (.017)*
Other/multi	− 1.41 (< .001)*
Poverty-income ratio	− 0.30 (< .001)*
Smoking	
Non-smoker	0.0 (Reference)
Current smoker	0.69 (.011)*
Physical activity	
No	0.0 (Reference)
Yes	− 1.89 (< .001)*
Fasting Insulin, pmol/L	0.03 (< .001)*

White: Non-Hispanic White; Mexican/Hispanic: Mexican or other Hispanic ethnicity; Black: non-Hispanic Black; Other/multi: other race or Multiracial

p-value calculated using GLM. Reference is the reference group used in the regression, so p-value not provided

*Significant at p-value of .05

National estimates based on complex survey design

In the logit portion of the two-part model, the likelihood of non-zero CRP based on fasting insulin, was statistically significant (OR = 1.02, CI 1.01, 1.03 p < .001), for every one-unit increase in fasting insulin the likelihood of a non-zero CRP increased by 2%. In the GLM part of this model, the adjusted association between fasting insulin was significantly associated with CRP (β = 0.03, CI 0.02, 0.04, p < .001). Although the beta estimates may appear small, it represents the increase in predicted CRP for each unit increase of insulin and is a clinically significant association. A fasting insulin of less than 60 pmol/L had a predicted CRP of 2.86 mg/L (CI 2.66, 3.06), 60–119 pmol/L had a predicted CRP of 4.39 mg/L (CI 3.98, 4.80), and a fasting insulin of 120 pmol/L or higher had a predicted CRP of 7.58 mg/L (CI 6.37, 8.79). The results from the reduced model including the predicted CRP based on each level of categorical variable are presented in Table 3.

**Table 3:** Reduced model: two-part model effects between fasting insulin and CRP among adults without diabetes

Covariate	Logit model		GLM model		Combine two-part model Predictive margins
	OR	p-value	β	p-value	Predicted CRP mg/L (CI)
Age, years	1.05	<.001	0.02	.013	
20–39					3.56 (3.26, 3.85)
40–59					4.07 (3.72, 4.42)
60–85					4.62 (4.03, 5.22)
Sex					
Male	Reference				3.78 (3.31, 4.25)
Female	0.83	.373	0.73	.001	4.22 (3.90, 4.53)
Race					
Non-Hispanic White	Reference				3.92 (3.57, 4.27)
Mexican/other Hispanic	2.29	.013	0.06	.851	4.41 (3.80, 5.03)
Non-Hispanic Black	0.75	.204	0.41	.149	4.72 (4.23, 5.21)
Other race/multi	0.51	.037	− 0.81	<.001	2.96 (2.51, 3.41)
Poverty-income ratio	1.02	.673	− 0.08	.168	
< 130% FPR					4.78 (4.35, 5.22)
130–349% FPR					4.18 (3.83, 4.53)
>=350% FPR					3.51 (3.17, 3.86)
Smoking					
Non/former smoker	Reference				3.83 (3.46, 4.21)
Current smoker	1.26	.280	1.26	<.001	4.62 (4.08, 5.17)
Physical activity					
No	Reference				5.19 (4.66, 5.72)
Yes	1.08	.757	− 0.86	.008	3.55 (3.21, 3.89)
Fasting Insulin, pmol/L	1.02	<.001	0.03	<.001	
< 60					2.86 (2.66, 3.06)
60–119					4.39 (3.98, 4.80)
≥ 120					7.58 (6.37, 8.79)

Mexican/other Hispanic: Mexican or other Hispanic ethnicity; Other race/multi: other race or Multiracial; CRP: C-reactive protein; OR: odds ratio; CI: confidence interval; FPR: federal poverty ratio

Reduced Model: controlling for age, sex, race, socioeconomic status (poverty-income ratio), smoking status, physical activity, and fasting insulin

Two-part regression model used; reference group is the reference group used for each categorical variable

Each level of categorical variable used to provide a predicted CRP based on the model

National estimates based on complex survey design

In the second model, which added waist circumference as a covariate, we found the odds of having a non-zero CRP based on fasting insulin was no longer statistically significant in the logit portion of the model (OR = 1.00, CI 0.99, 1.00 p = .307), while in the GLM portion of the model the association between fasting insulin and CRP remained significant (β = 0.01, CI 0.002, 0.02, p = .012). The predicted CRP based on category of fasting insulin in the model, was 2.94 mg/L (CI 2.74, 3.14) for those with a fasting insulin of less than 60 pmol/L, 4.42 mg/L (CI 4.05, 4.79) for those with a fasting insulin between 60 and 119 pmol/L, and 6.39 mg/L (CI 5.28, 7.50) for those with a fasting insulin of 120 pmol/L or more demonstrating the clinical significance of this relationship. The relationship between the highest level of fasting insulin and CRP is attenuated in the full model controlling for waist circumference. These results are presented in Table 4. The predicted CRP for each level of each categorical variable are presented in Table 4.

**Table 4:** Full model: two-part model effects between fasting insulin and CRP among adults without diabetes

Covariate	Logit Model		GLM Model		Combine two-part model Predictive margins
	OR	p-value	β	p-value	Predicted CRP mg/L (CI)
Age, years	1.02	.002	0.01	.015	
20–39					3.45 (3.17, 3.73)
40–59					4.01 (3.70, 4.33)
60–85					4.40 (3.98, 4.82)
Sex					
Male	Reference		Reference		3.67 (3.23, 4.11)
Female	1.62	.046	0.96	<.001	4.09 (3.81, 4.36)
Race					
Non-Hispanic White	Reference		Reference		3.76 (3.45, 4.08)
Mexican/other Hispanic	2.12	.027	0.38	.241	4.30 (3.62, 4.99)
Non-Hispanic Black	0.83	.447	0.41	.094	4.54 (4.07, 5.01)
Other race/multi	0.81	.528	0.11	.620	3.32 (2.87, 3.76)
Poverty-income ratio	1.01	.835	− 0.14	.004	
< 130 FPR					4.63 (4.20, 5.06)
130–349% FPR					4.09 (3.77, 4.41)
>=350% FPR					3.39 (3.09, 3.69)
Smoking					
Non/former smoker	Reference				3.71 (3.39, 4.03)
Current smoker	1.40	.121	1.22	<.001	4.50 (3.88, 5.12)
Physical activity					
No	Reference				4.87 (4.39, 5.34)
Yes	1.07	.796	− 0.52	.050	3.52 (3.21, 3.83)
Waist circumference, cm	1.13	<.001	0.07	<.001	
Females <=88.9 cm (35 in)					2.53 (2.25, 2.82)
Females > 88.9 cm (35 in)					5.11 (4.75, 5.47)
Males <=101.6 cm (40 in)					2.62 (2.31, 2.93)
Males > 101.6 cm (40 in)					4.93 (4.28, 5.58)
Fasting Insulin, pmol/L	1.00	.307	0.01	.012	
< 60					2.94 (2.74, 3.14)
60–119					4.42 (4.05, 4.79)
≥ 120					6.39 (5.28, 7.50)

Mexican/other Hispanic: Mexican or other Hispanic ethnicity; Other race/multi: other race or Multiracial; CRP: C-reactive protein; OR: odds ratio; CI: confidence interval; FPR: federal poverty ratio

Full Model: controlling for age, sex, race, socioeconomic status (poverty-income ratio), smoking status, physical activity, waist circumference and fasting insulin

Two-part regression model used; reference group is the reference group used for each categorical variable

Each level of categorical variable used to provide a predicted CRP based on the model

National estimates based on complex survey design

## Discussion

This analysis found that fasting insulin was associated with CRP which remained while controlling for waist circumference. In the reduced model, which did not control for waist circumference, both the odds of having an CRP higher than 0.1 mg/L (OR = 1.02, p < .001) and the association between fasting insulin and CRP in the GLM model (β = 0.03, p < .001) were statistically significant. In the full model which also controlled for waist circumference, the logit portion of the model was no longer significant (OR = 1.00, p = .307), and statistical significance was maintained in the GLM (β = 0.01, p = .012), although the relationship was attenuated for the highest level of insulin. Furthermore, the predicted CRP based on category of fasting insulin in the full model which also controlled for waist circumference, was 2.94 mg/L in the lowest tertile fasting insulin, 4.42 mg/L for those in the moderate tertile, and 6.39 mg/L in the highest tertile.

The present results confirm a previous report of the association between insulin and CRP while controlling for waist circumference [13]. Both our and Meng et al. (2007) studies support the conclusion that this relationship between fasting insulin and inflammation is independent of waist circumference. Additionally, our full model illustrates that while all tertiles predict a CRP above the American Heart Association guidelines stating that an hs-CRP of 2.0 or higher is an atherosclerotic cardiovascular disease (ASCVD) risk enhancer, the risk increases with increased fasting insulin level [36].

The association between insulin and inflammation may have implications for treatment of those with T2DM. A direct association between CRP and both insulin use and dose were found in participants with a body mass index (BMI) of less than 30, but not in obese participants [14]. Current T2DM treatment guidelines recommend specific glycemic targets without consideration for the treatments’ effect on insulin. Many glucose-lowering medications increase insulin levels such as sulfonylureas and exogenous insulin, while other therapies decrease insulin requirements such as metformin [37] and sodium-glucose cotransporter 2 inhibitors (SGLT-2s) [18]. Other medications such as glucagon-like peptide-1 receptor agonists (GLP-1s) have mixed effects as they increase postprandial insulin but may decrease insulin secretion over time due to effects on appetite, weight, and insulin resistance [38, 39]. Current medication guidelines recommend metformin as the first line of treatment [19, 40]. Additionally, SGLT-2 or GLP-1s are recommended for patients with established or at high risk for atherosclerotic cardiovascular disease [40] and have been found to decrease mortality and cardiovascular disease in people with type 2 diabetes [40].

Medications such as metformin [41] and SGLT2s [18] have cardiovascular benefits that are not attributed to the decrease in HbA1c. Controlling HbA1c is more effective at preventing microvascular complications such as nephropathy and retinopathy, than macrovascular outcomes such as cardiovascular disease [18]. Metformin and SGLT-2s improve many markers beyond HbA1c, including hyperinsulinemia [18, 41, 42]. Additionally, GLP-1s improve insulin-sensitivity [39] which may decrease hyperinsulinemia over time. GLP-1s exhibit anti-inflammatory effects, although the mechanism by which this occurs is still being investigated [38]. If higher insulin levels are associated with higher levels of inflammation, using therapies such as SGLT-2s that acutely decrease insulin levels and GLP-1s that may decrease insulin levels over time, while simultaneously lowering glucose levels, should be recommended for obese people with T2DM if additional pharmacotherapy is needed in addition to, or in place of, metformin. Based on the promising effects from medication trials, dietary strategies capable of simultaneously decreasing insulin and glucose levels may have similar positive cardiovascular benefits. However, future studies are need to ascertain if the choice of pharmacotherapy, namely those that decrease insulin levels, decreases CRP or other markers of chronic inflammation in this population.

Limitations of the present study include its cross-sectional nature and consequent inability to determine causality. The interplay between insulin and inflammation and between insulin, inflammation, and diabetes is complex and likely influenced by metabolic dysfunction, and therefore, the results of the present study may not generalize to a population of adults with T2DM. Additionally, we were not able to control for all medications which may decrease inflammation due to the lower response rates and the inability to distinguish between daily and sporadic use on this section of the survey. Despite these limitations, this study does provide insight into the association between fasting insulin and CRP in adults without diabetes.

This study found an association between fasting insulin and CRP that persisted after controlling for waist circumference, demonstrating a relationship independent of adiposity. These results suggest that treatment approaches that simultaneously decrease insulin levels as well as glucose levels may provide additive anti-inflammatory effects, and therefore may improve long-term outcomes for adults with type 2 diabetes.

## Data Availability

The datasets generated and analyzed during the current study are publicly available on the website https://www.cdc.gov/nchs/nhanes/index.htm.
